# Effect of Different Pig Fecal Microbiota Transplantation on Mice Intestinal Function and Microbiota Changes During Cold Exposure

**DOI:** 10.3389/fvets.2022.805815

**Published:** 2022-04-13

**Authors:** Ting Liu, Yanbo Guo, Chang Lu, Chunbo Cai, Pengfei Gao, Guoqing Cao, Bugao Li, Xiaohong Guo, Yang Yang

**Affiliations:** Department of Animal Sciences, Shanxi Agricultural University, Taigu, China

**Keywords:** gut microbiota, absorptive capacity, gut microbiota cold exposure, microbiome, cold exposure, fecal microbiota transplantation, Mashen pigs

## Abstract

Cold stress influences intestinal processes, causing physiological and immunological responses in animals. Intestinal microbiota participates in maintaining the stability of the intestinal environment. However, phenotypic characteristics and the effects of porcine microbiota changes under cold conditions remain poorly understood. Here, the fecal microbiota of cold tolerant breed (Mashen) and cold sensitive breed (Duroc-Landrace-Yorkshire) was transferred to germ-free mice, respectively. After a cold exposure (4°C) for 21 days, intestinal function and microbe changes of mice were explored. The results showed that Mashen pigs microbiota transplantation made the body temperature of the mice stable, in which the fat weight and expression of uncoupling protein 1 (*UCP*1), carnitine palmitoyltransferase 1B (*Cpt*1b), and Peroxisome proliferator-activated receptor-gamma coactivator (*PGC-*1α) were significantly higher (*P* < 0.05) than those of the control group. The results of intestinal structure and expression of serum inflammatory factors showed that fecal microbiota transplantation (FMT) mice have more intact intestinal structure and high expression of proinflammatory factor such as interleukin-4 (IL-4). The study of mice fecal microbiome characterized *via* 16S rRNA sequencing found that pig microbiota transplantation changed the abundance of Firmicutes. In addition, it identified discriminative features of Firmicutes in the microbiota between two breeds of pig, in which *Clostridiaceae* were enriched in the microbiota community of Mashen pig and *Coriobacteriales* were significantly (*P* < 0.05) enriched in the Duroc-Landrace-Yorkshire pig microbiota transplantation group based on linear discriminant analysis effect size (LEfSe) analysis. Finally, we found that the content of propionic acid and butyric acid in rectal contents significantly changed and the abundances of *Clostridium* and *Lachnospira* showed significant correlations with changes in short-chain fatty acids. The results suggest that pig fecal microbiota transplantation can alleviate the changes in physiological and biochemical indicators in mice caused by cold exposure. Mice have gut microbes altered and improved gut barrier function via fecal microbiota transplantation in pigs.

## Introduction

In developing countries, livestock plays an important role in agricultural production. Animals are subjected to drastic changes in climate and will produce a series of pathological reactions. Therefore, study on mitigating the adverse effects of extreme climates on livestock remains explored (1). Pigs experience various stressors throughout their lifetime. The thermoneutral zone for most mammals varies with age and body weight (2). For young growing pigs, this is particularly important as they lack significant sources of adipose at birth and do not develop these stores until maturity (3). Thermal stress imposed by fluctuations in environmental temperature affects animal physiological, biochemical, and molecular signal regulation (4, 5). The exposure of mammals to temperatures below their thermoneutral threshold (21°C for pigs, 30°C for mice, and 24°C for humans) is regarded as a cold stimulus (6). Liu's group found that, compared to mice in normal (23°C) temperature, cold stress induces a significant decrease in the core body temperature of mice (7). Notably, cold exposure induces metabolic parameter-associated alterations in the gut microbiota (8). From 2016 to 2018, Liu et al. did a comprehensive analysis on the cold tolerance of Min pigs. With the rapid development of molecular biology technology, the analysis and study of the genetic basis of the cold resistance characteristics of pigs have been possible from the perspective of molecular genetics (9). Their study preliminarily validated the cold resistance characteristics of landrace pigs. However, systematic studies of gut microbial changes in relation to cold tolerance have not been performed.

The gastrointestinal tract is the largest endocrine organ in the body that releases several regulatory peptide hormones that influence many physiological processes (10). The animal gut consists of physical barriers, chemical barriers, immune barriers, and biological barriers that work together to maintain gut health. The microorganisms that inhabit the gastrointestinal tract of mammals, termed the gut microbiota, are critical in host physiology regulation by influencing metabolism, inflammation, aging, and behavior (11). The gut microbiota contributes to energy homeostasis and microbial colonization of the gut begins directly after birth and develops in response to genetic and environmental factors (12). The gut microbiota and the intestinal mucosal system co-evolve and the host provides a hospitable environment and nutrients, allowing the gut microbiota to shape immune system development and function (13, 14). The small intestine surface is characterized by long finger-like projections called villi, which become progressively shorter and broader down the small intestine. The intestinal epithelium is the major site of adaptive immune cell accumulation within the intestine (15). A balanced regulation between proinflammatory and anti-inflammatory cytokines responses in the gastrointestinal tract leads to protection against pathogens and tolerance to stress (16). Kaushik and Kaur (17) exposed rats at 6–8°C for 3 weeks, resulting in intestinal inflammation in rats. Recent studies in mice indicate that decreasing the housing temperature alters the gut microbiota (18). Cold exposure affects gut morphology, function, and composition of gut microbes.

Fecal microbiota transplantation (FMT) can provide insight into host–microflora interactions, characteristics, and understanding of disease physiology and pathology. C57BL/6J mice have the advantages of a long life cycle and easy feeding and are often used in experimental animal models of physiology and pathology (19), thus facilitating the examination of microbiota composition changes following colonization in various settings. The use of fecal transplantation technology can effectively demonstrate the physiological characteristics of the donor and discover how much the donor characteristics can be lost with the transfer (20). Therefore, gut microbiota transplantation may demonstrate the important role of microbes in some physiological and pathological processes. In our experiments, gut microbiota transplantation could demonstrate the effects of different pig breeds on thermogenic modifications in mice.

Duroc-Landrace-Yorkshire (DLY) pigs, commonly used commercial pigs, are susceptible to ambient temperature, significantly reducing their feed intake, and enhancing the production of immune proteins in cold environments (21). Compared to DLY pigs, Mashen pigs show stronger cold resistance as they grow in cold regions (22–24). In the early stage of the experiment, we conducted a cold stress test on Mashen pigs and other breeds of pigs and found that Mashen pigs had better health status and smaller changes in body temperature. We found that the bacterial microbiome of Mashen pigs and other pig breed is very different and the bacterial flora has a certain impact on the growth performance of pigs (25, 26). However, whether the difference of microbes influencing cold resistance or not remains unclear. Therefore, in this study, we transplanted fecal bacteria into mice and placed them at 4°C for 21 days. By comparing the physiological indicators and intestinal microbes of the mice in different microbiota transplantation, we explored difference in cold tolerance between Mashen (MS) pigs and Duroc-Landrace-Yorkshire (DLY) pigs. Our data could provide initial validation for further study into the role of the pig microbiome in cold environments.

## Materials and Methods

### Inoculum Preparation for Fecal Microbiota Transplantation

All the animal procedures were performed in strict accordance with the Code of Ethics of the World Medical Association (https://www.wma.net/policies-post/wma-statement-on-animal-use-in-biomedical-research/). The management and design of the experiment were kept to animal care rules approved by the Animal Ethics Committee of Shanxi Agricultural University (Shanxi, China). For fecal microbiota transplantation (FMT), collected in the early morning of the first day of feeding, from five, 2-month-old Mashen pigs (MSt) and five, 2-month-old Duroc-Landrace-Yorkshire pigs (DLYt) 20 mg feces from each pig, all the feces were mixed, then 5 ml sterile water was added, mixed with a magnetic stirrer, then divided into 6 portions, filtered through an autoclaved metal sieve, and added 5 ml of 10% glycerol. The suspension was stored at −80°C until use. 200 μl of the suspension was then gavaged to each FMT mouse, *n* = 7 per group (27). For the control (Ctrl) group (*n* = 7), saline (200 μl) was delivered by intragastric gavage to each mouse.

### Fecal Microbiota Transplantation and Treatments

Experiments were employed on 21 6-week-old male C57BL/6J [specific pathogen free (SPF) type] mice (SPF Biotechnology Ltd., Beijing, China), with each mouse housed in a single cage. Mice were kept in a specific pathogen-free facility in 12 h day/night cycles in individually ventilated cages (28). Acclimatized animals were allocated to the experimental groups based on their body weight levels to ensure equal starting points. For the depletion of microbiota, fresh antibiotics (200 mg/kg ampicillin, neomycin and metronidazole, and 100 mg/kg vancomycin) were administered once a day at 9 a.m. in 500 μl *via* intragastric gavage for 7 days, as described previously (12, 19). At the end of 7 days, all the mice were treated with 200 μl by polyethylene glycol-recipients (PEG-r). Polyethylene glycol (PEG) solution contained PEG 4000 (Orion Pharma, Espoo, Finland) (77.5 g/l), sodium chloride (1.9 g/l), sodium sulfate (7.4 g/l), potassium chloride (0.98 g/l), and sodium bicarbonate (2.2 g/l) were diluted in sterile tap water (19). Fecal microbiota transfer was performed 24 h after the PEG administration.

Fecal microbiota transplantation was given twice a day for 3 days. Cold exposure was performed 1 day after fecal microbiota transplantation. The ambient temperature of the mice was adjusted to 4°C. The cold treatment was carried out for 21 days. During the period, the mice received sterilized food and water *ad libitum*. The feed was from the same supplier and batch, the water was distilled, and the feed and water were placed in glass containers daily under autoclave for 2 h method for sterilization.

### Body Weight and Rectal Temperature

Mice were weighed every 3 days from the beginning (day 1) to the end of day 21 of the cold exposure by using an electronic balance (±0.1 g; BL 1500; Sartorius, Göttingen, Germany) and body weight change was calculated. We measured rectal temperature in mice that were housed at ambient temperature by using a medical precision thermometer at 20:00 p.m. every 3 days and we measured ambient temperature before cold stress in mice. Body temperature was tested after the body weight testing (the body temperature taken while they were in the cold climate), by inserting a mercury thermometer into the rectum of the mice for 5 min, and each mouse was sterilized by 70% alcohol disinfection around the anus before the body temperature test. The thermometer was sterilized between the temperature tests of each mouse.

### Sample Collection, Blood Profiles and Morphological Observations

After 21 days of cold exposure, mice were anesthetized, blood was drawn, and mice were euthanized with 6% CO_2_ for 10 min. The skin of the mouse was scrubbed several times with 75% alcohol and then the skin on the neck was lifted and peeled off the skin, separate it from the muscle and fat tissue. Neck subcutaneous adipose was separated (29) and weighed with an analytical balance. Intestinal tissue samples were then collected. The rectal fecal samples were removed on a horizontal flow clean bench. All the samples were frozen immediately in liquid nitrogen and stored at −80°C. A complete blood count (CBC) was used in mice for a blood test. Blood samples were centrifuged (5810R; Eppendorf, Hamburg, Germany) for 15 min at 3,000 rpm at 4°C. The sera were carefully moved into plastic vials and stored at −20°C. The concentrations of interleukin-4 (IL-4), interleukin-6 (IL-6), and tumor necrosis factor-α (TNF-α) were analyzed by ELISA (ELISA Starter Accessory Package, mouse ELISA Mlbio, Shanghai). Ileum and jejunum tissues were fixed in formalin (3.7% in 90% ethanol) for 24 h and subsequently embedded in paraffin. After tissue dehydration and clearing, tissue was cut in 5 μm thick sections and stained with H&E and periodic acid-Schiff (PAS)-alcian blue using standard techniques. Image Analysis—ProPlus version 6.0 (Media Cybernetics Incorporation, USA) software measured villus length, recess depth, and calculated villus length to recess depth (V/C) ratio (30).

### Real-Time PCR

For RNA expression analysis, total RNA from ~30 mg of frozen adipose and intestinal samples was extracted using Trizol (Life Technologies) and resuspended in nuclease-free water (Life Technologies). A 1–2 μg sample of total RNA was used for reverse transcription using the QuantiTect Reverse Transcription Kit (Qiagen NV). Then, complementary DNA (cDNA) (10X diluted) was used for quantitative PCR (qPCR) using the Power SYBR Green PCR Master Mix (Life Technologies) and real-time PCR (7300 HT, Applied Biosystems). Primer sets for qPCR analyses are shown in Supplementary Table 1.

### Microbiota DNA Extraction and 16S rRNA Sequencing

Microbial DNA was extracted from mice rectal fecal samples (*n* = 4/group) by using the fecal genomic DNA extraction (DP712, Norcross, Georgia, USA) according to the manufacturer's protocols (31). The final DNA concentration and purification were determined using the NanoDrop 2000 UV-Vis spectrophotometer and DNA quality was checked by 1% agarose gel electrophoresis (Thermo Fisher Scientific, Waltham, Massachusetts, USA). The V3–V4 hypervariable regions of the bacteria 16S rRNA gene were amplified with primers 338F (5′-ACTCCTACGGGAGGCAGCAG-3′) and 806R (5′-GGACTACHVGGGTWTCTAAT-3′) by using the thermocycler PCR system [GeneAmp 9700, ABI, USA; (32)]. The resulting PCR products were extracted from 2% agarose gel, further purified using the AxyPrep DNA Gel Extraction Kit (Axygen Biosciences, Union City, California, USA), and quantified using QuantiFluor™-ST (Promega, USA) according to the manufacturer's protocol. Purified amplicons were pooled in equimolar and paired-end sequenced (2 × 300 bp) on an Illumina MiSeq platform (Illumina, San Diego, USA). Sequencing reads containing more than 10% ambiguous bases (N) or with sequence qualities below 80% (Q-value > 20) were filtered from raw data. Filtered reads were subsequently merged using Flash software with a minimum overlap length of 10 bps and maximum allowed mismatch ratio of 0.2 in overlapping regions. These merged reads were then subjected to quality control and data optimization using Trimmomatic software (33). The reads were available in the National Center for Biotechnology Information (NCBI) Slum Rehabilitation Authority (SRA) database under BioProject accession number PRJNA734723.

### Short-Chain Fatty Acids

Six short-chain fatty acids (SCFAs), including acetic, propionic, butyric, isobutyric, valeric, and isovaleric acid, were measured in rectal fecal samples by high-performance gas chromatography (34) with a gas chromatography (GC) autosampler and flame ionization detector (FID) system following a modified method (35, 36). Rectal contents were extracted directly with water without derivatization (36). Separations were performed in a 30 m × 0.25 mm × 0.25 μm DB-WAX column (Agilent Technologies) by using 99.998% hydrogen as carrier gas at a flow rate of 1.0 ml/min. The system was operated at 250°C. Injections were performed in the splitless mode at 230°C and 0.5 μl of sample was used for each injection. The oven temperature was programmed from 60 (1 min) to 200°C at 5°C/min and then from 200 to 230°C at 10°C/min. The total running time of each sample was 32 min.

### Statistical Analysis

SPSS version 23.0 software (https://www.ibm.com/cn-zh/analytics/spss-statistics-software) was used to analyze the results of mouse body weight, body temperature, blood count, intestinal H&E-stained slices, PAS-stained slices, qPCR, ELISA, and SCFA tests. Duncan's multiple range test was used for comparison between the groups. To compare the diversity index of gut microbes between the two groups, the Wilcoxon rank sum test was performed with Vagen software package (version 2.5.3) in R version 3.3.2 (31), beta diversity analysis was performed by Welch's *t*-test and Wilcoxon rank sum test with Vagen software, and linear discriminant analysis effect size (LEfSe) software was used to screen the biomarker characteristics of each group to analyze the effect size. Functional prediction of operational taxonomic units (OTUs) was performed using the Tax4Fun software (version 1.0). Interclass principal component analysis (PCA) was calculated and statistically assessed using the Monte Carlo rank test to observe the net effect on microbiota scatter in different mice. Venn diagrams and heatmaps were generated using R's Venn diagram and superheat packages, respectively. Bar, scatter, box, and whisker plots were generated using GraphPad Prism version 7.0® (https://www.graphpad.com/scientific-software/prism/). Adjusted *p* < 0.05 were considered as statistically significant.

## Results

### Phenotypes Differed Among the Three Groups

**Figure 1:**
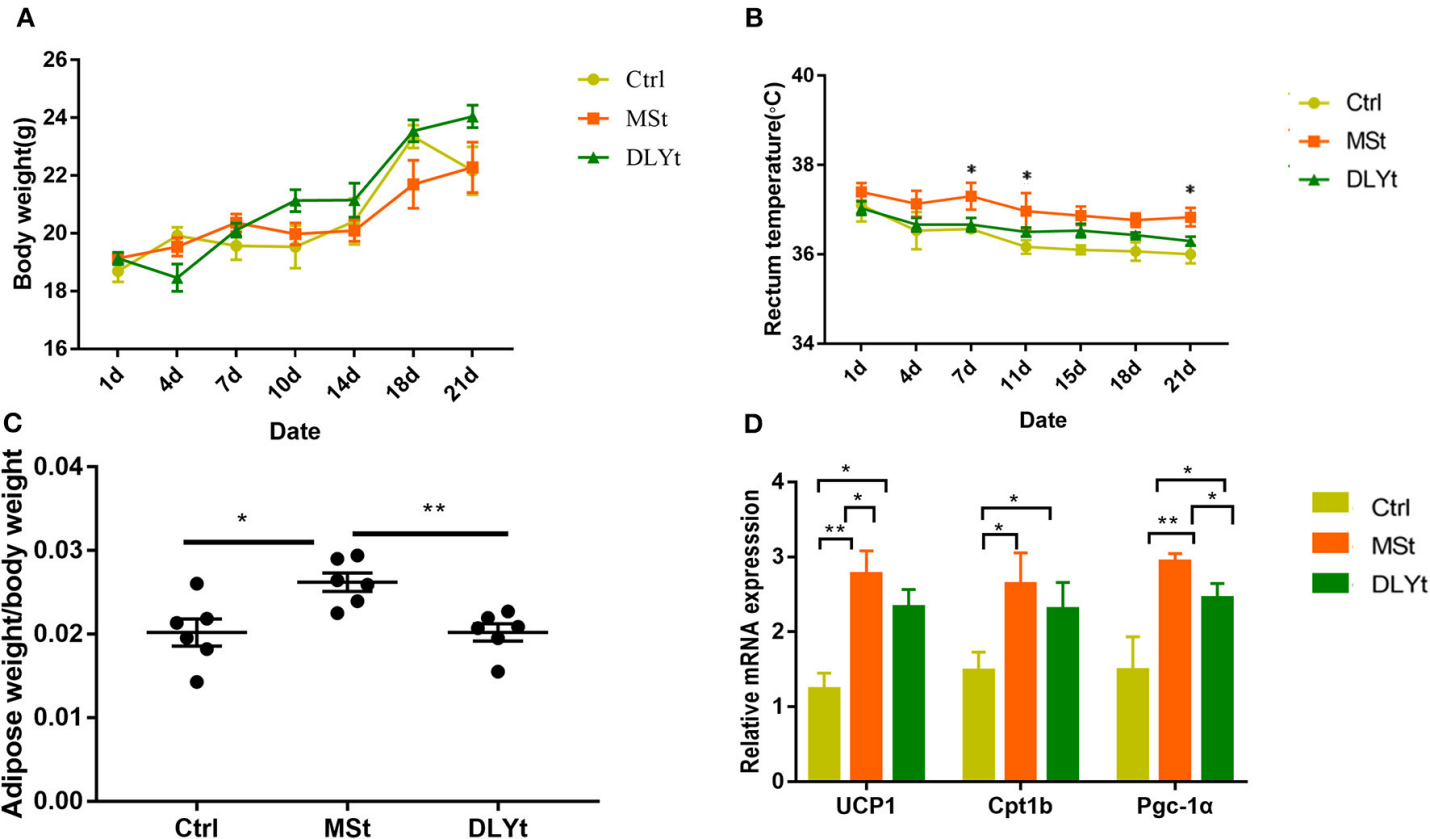
Effect of fecal microbiota transplantation (FMT) on metabolic phenotypes and general immunity during cold exposure. **(A–C)** Body weight, body temperature (*n* = 7/group), and adipose weight/body weight rate in FMT and control mice during cold exposure. **(D)** Results of relative gene messenger RNA (mRNA) of adipose thermogenesis (*n* = 7/group). (*P < 0.05, **P < 0.01, and ***P < 0.001, Duncan's multiple range test). *** means extremely significant.

Body weight after acclimation (end of cold exposure day 21) was not affected by cold (Figure 1A). MSt mice tended to gain less body weight than that of DLYt mice and control (Ctrl) mice, but the difference was not significant. Rectal temperature of mice before cold exposure was 37.5 ± 1.2°C and cold exposure for 21 days led to more stable body temperature in MSt than that of DLYt mice and Ctrl mice (Figure 1B). We observed that the adipose weight in MSt mice was higher than that in control mice (*P* < *0.0*5), and DLYt mice (*P* < 0.01, Figure 1C), but no significant difference was observed in intestinal weight (Supplementary Figure 1). In addition, the expression of adipose thermogenic genes such as *UCP*1 in FMT mice was significantly (*P* < 0.05) higher than that in the control group. The expression of *UCP*1 and *PGC-*1α genes in MSt mice was significantly higher than that in the control group (*P* < 0.05, Figure 1D). The number of leukocytes in Ctrl mice was significantly higher than that of MSt mice and DLYt mice (Supplementary Figure 2). Therefore, FMT changes mouse rectal temperature, intestinal length, and adipose weight.

### Mice Intestinal Morphology Was Reshaped by Fecal Microbiota Transplantation Under Cold Exposure

**Figure 2:**
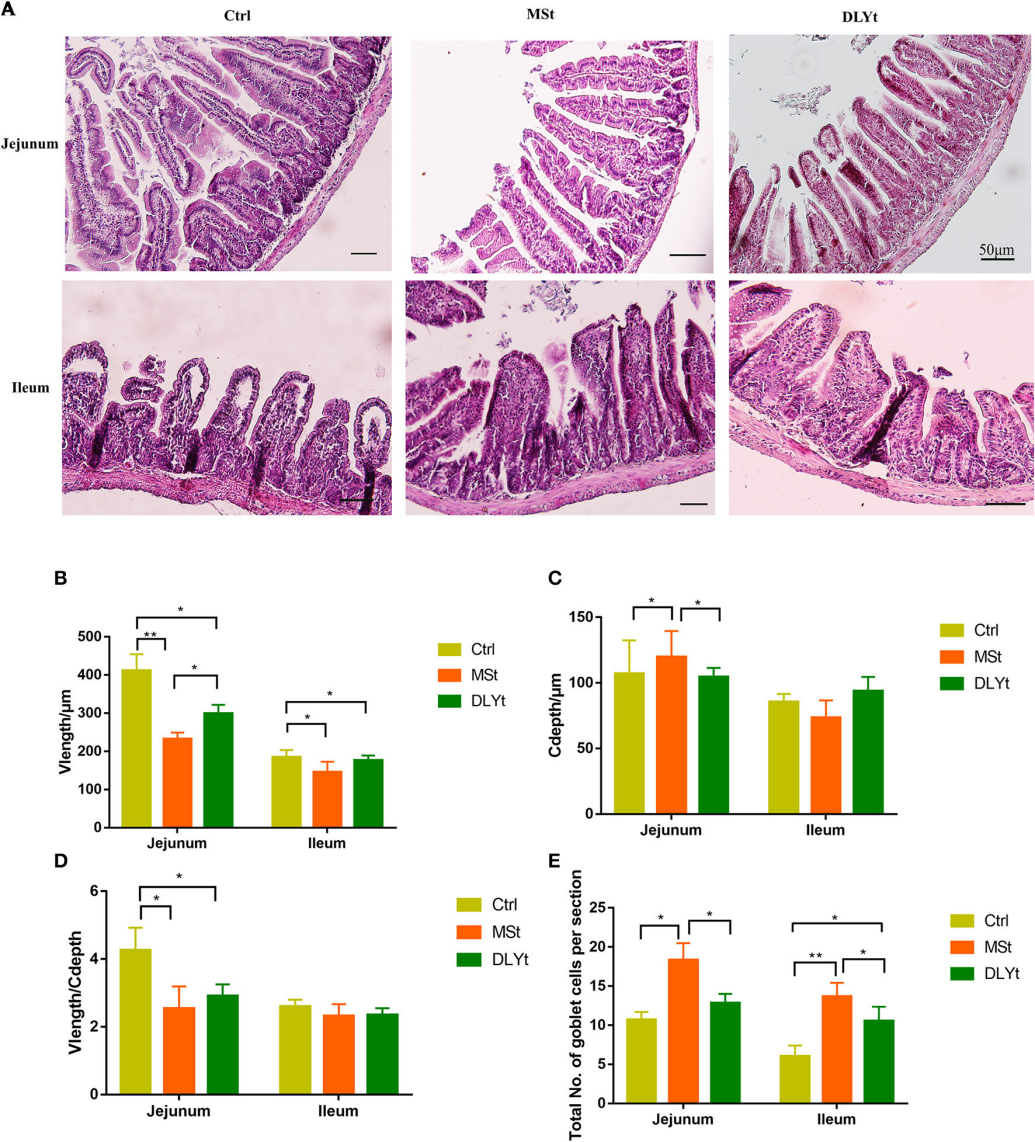
Effect of FMT on intestinal form, cell composition, and immune factors during cold exposure. **(A)** The ileum intestinal tissue H&E-stained sections of control, the Mashen pigs (MSt) and Duroc-Landrace-Yorkshire pigs (DLYt) group (*n* = 7/group), and the jejunum intestinal tissue H&E-stained sections of the control, the MSt and DLYt group (*n* = 7/group). **(B–E)** Results of intestinal morphology of each group. Detection results of intestinal goblet cell number (*n* = 7/group). (*P < 0.05, **P < 0.01, and ***P < 0.001, Duncan's multiple range test). *** means extremely significant.

Intestinal morphology observation showed that the villi of FMT mice were arranged neatly with fewer damages and breaks (Figure 2A). To further investigate the changes in the intestinal morphology, we measured the villus length (Figure 2B) in jejunum and ileum and found that the villus length in jejunum of MSt mice and DLYt mice were significantly (*P* < 0.05) shorter than those of Ctrl mice and MSt mice were significantly (*P* < 0.05) shorter than DLYt mice and the villus length in ileum of MSt mice and DLYt mice were significantly (*P* < 0.05) shorter than those of Ctrl mice. By detecting the recesses depth (Figure 2C), we found that recesses depth in jejunum of MSt mice was significantly (*P* < 0.05) higher than those of Ctrl mice and DLYt mice. Measuring the ratio of villus length and recesses depth (V/C) (Figure 2D), we found that V/C in jejunum of Ctrl mice was significantly (*P* < 0.05) higher than those in MSt mice and DLYt mice. FMT mice intestinal villus length was shorter than Ctrl mice and this change may be caused by fecal bacteria transplantation (Supplementary Table 3). To investigate the intestinal morphological changes, we quantified the goblet cells (Figure 2E, Supplementary Figure 2). By detecting the goblet cells per section, it was found that in jejunum, MSt mice had significantly (*P* < 0.05) higher goblet cells than Ctrl mice and DLYt mice. In ileum, goblet cells of FMT mice were significantly (*P* < 0.05) higher than Ctrl mice and goblet cells of MSt mice were significantly (*P* < 0.05) higher than DLYt mice.

### Expression of Inflammation Was Altered by Fecal Microbiota Transplantation Under Cold Exposure

**Figure 3:**
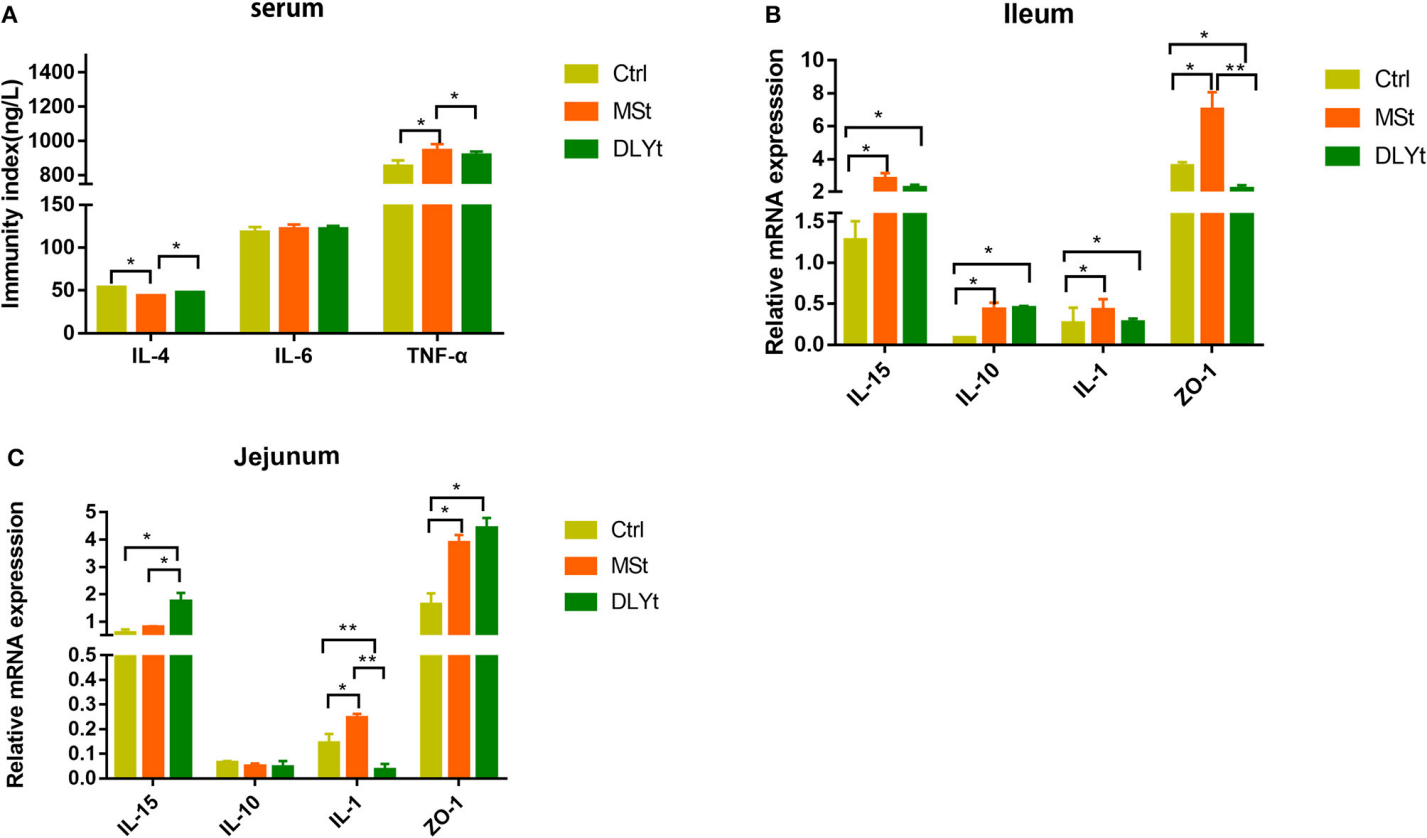
Effect of FMT on immune factors during cold exposure. **(A)** Interelukin-4 (IL-4), interleukin-6 (IL-6), and tumor necrosis factor-α (TNF-α) levels (*n* = 7/group) in serum. *P < 0.05. **(B,C)** Results of relative gene mRNA of jejunum **(B)** and ileum **(C)** (*n* = 7/group). (*P < 0.05, **P < 0.01, and ***P < 0.001, Duncan's multiple range test). *** means extremely significant.

We further measured several cytokines, including IL-4, IL-6, and TNF-α in serum by ELISA, and did not detect significant difference in IL-6 between the control and FMT groups. Notably, the control mice had higher expression in IL-4 and lower expression in TNF-α than MSt mice (Figure 3A). The messenger RNA (mRNA) expression of IL-15 was significantly higher in DLYt mice than in Ctrl and MSt mice (Figure 3B). By detecting the expression of related mRNAs, the results showed that in the ileum, the mRNA expression of IL-15 in DLYt mice was significantly higher than that in Ctrl mice and MSt mice (Figure 3B); the expression levels of zonula occludens-1 (*ZO-*1) relative mRNA of MSt mice showed higher expression (*P* < 0.05) than those of DLYt mice and Ctrl mice. In the jejunum, the expression levels of relative mRNA differed and MSt mice showed higher expression of *IL-*15 and *ZO-*1 than the Ctrl mice (Figure 3C). Together, these data suggest that FMT alters inflammatory cytokines during cold exposure.

### Microbial Diversity and Composition Differences in Fecal Microbiota Transplantation Mice

**Figure 4:**
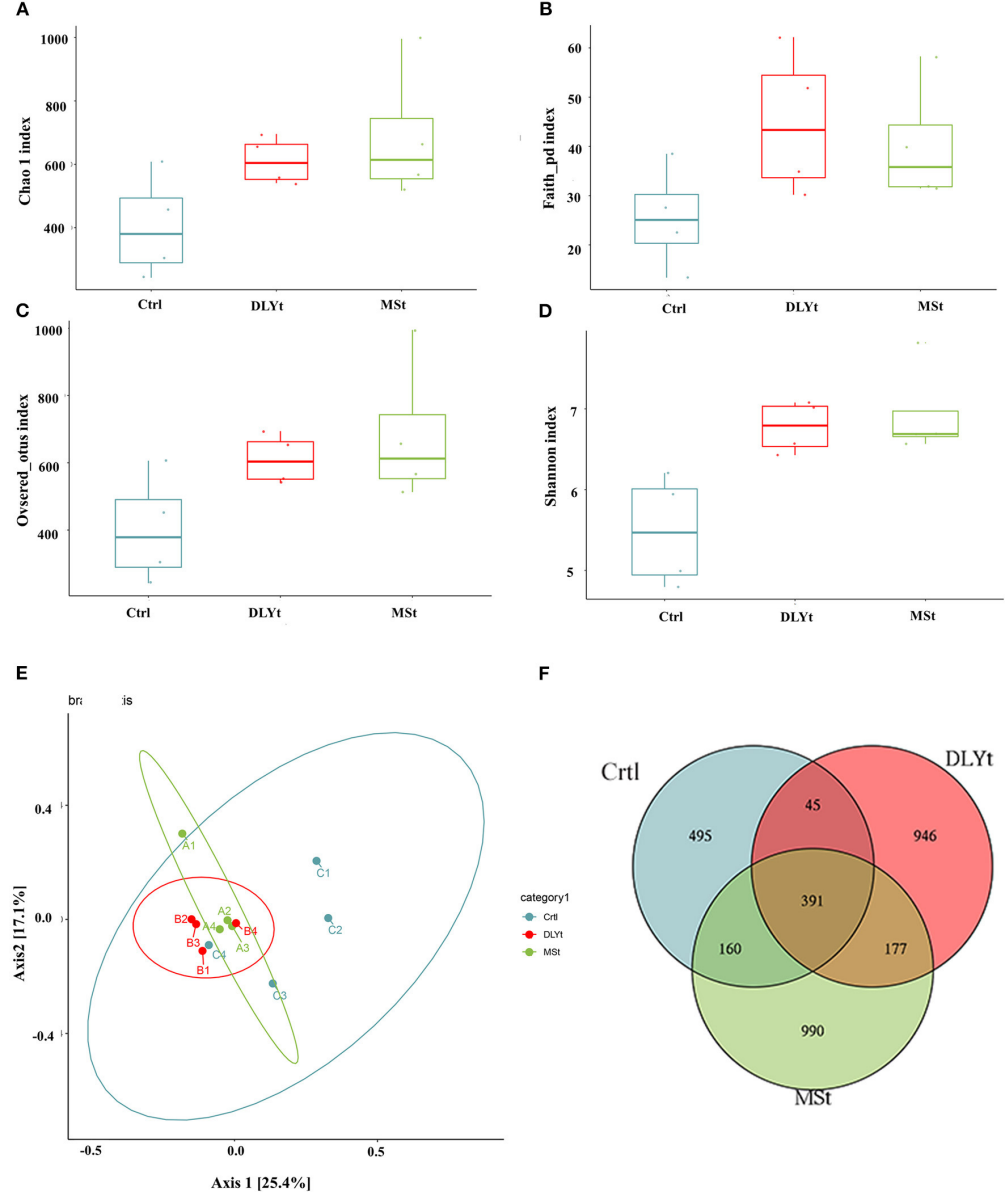
FMT altered the diversity of fecal microbiota (*n* = 4/group). **(A–D)** Analysis results of alpha diversity index (Wilcoxon rank sum test). **(E)** Principal coordinate analysis plots based on weighted UniFrac distance. Each symbol represents a single sample of fecal contents from the three groups (Welch's *t*-test and Wilcoxon rank sum test). **(F)** Venn diagram of the number of intestinal genera in the fecal samples from the three groups.

To identify potential mechanisms for FMT influencing mice cold resistant, we used 16S rRNA-seq to identify the characteristic of microbes. A total of 1,194,054 reads with an average length of 101,557 bps remained after quality filtering and these reads were categorized into 3,204 OTUs. The alpha (α) diversity of fecal microbiota from control, Mst mice, and DLYt mice was determined on the basis of Chao1 index (Figure 4A), PD index (Figure 4B), observed_OTUs index (Figure 4C), and the Shannon index (Figure 4D). The results showed that the alpha diversity of the microbiota of FMT mice is higher than that of control mice (*P* = 0.08). There was no significant difference between the DLYt mice and MSt mice in the alpha diversity of the microbiome. Principal coordinate analysis (PCoA) demonstrated beta diversity across the three groups, indicating moderate clustering of samples (Figure 4E). Venn diagram showed that there were 946 unique genera in DLYt mice and 990 unique genera in MSt mice (Figure 4F).

### Fecal Microbiota Transplantation Changes Fecal Microbiota Diversity in Mice Under Cold Exposure

**Figure 5:**
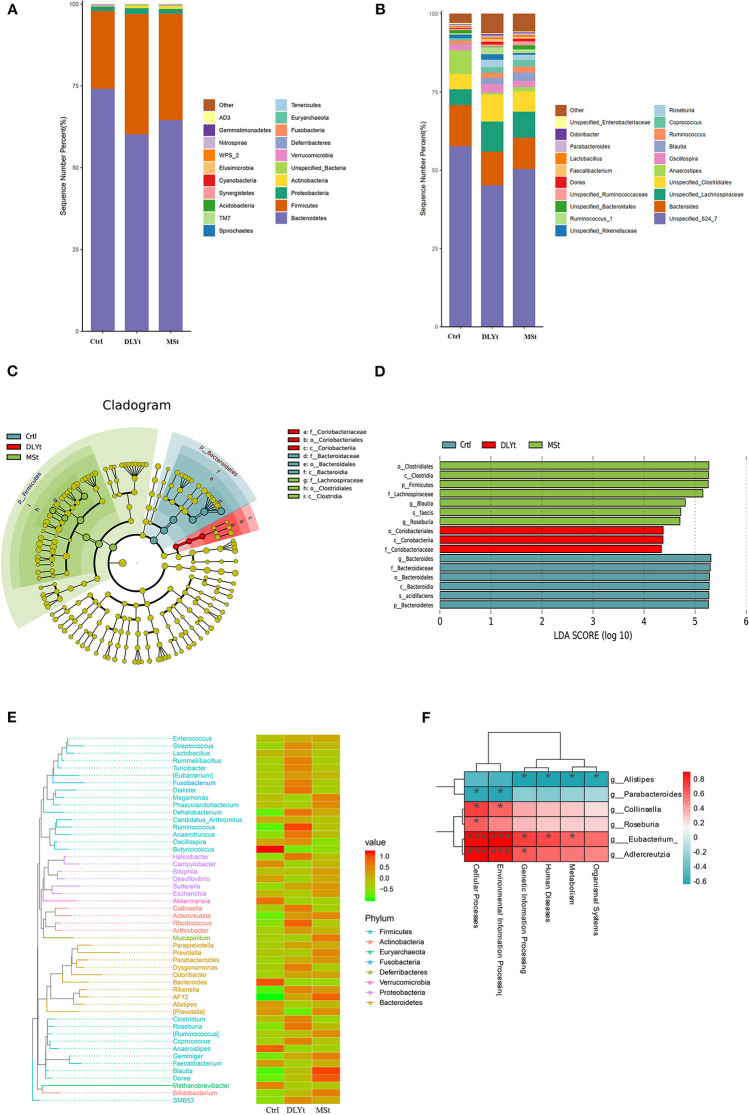
FMT altered the composition of fecal microbiota. **(A,B)** Relative abundance at the phylum and family levels in fecal microbiota community of the three groups. **(C)** Cladogram representing the taxa enriched in fecal microbiota community of the four groups detected by the linear discriminant analysis effect size (LEfSe) tool. Differences were represented by the color of the most abundant class (red indicating the DLYt group, green indicating the MSt group, and yellow indicating nonsignificant). The diameter of each circle is proportional to the taxon's abundance. **(D)** Differential bacterial taxonomy selected by LEfSe analysis with LDA score > 4 in the fecal microbiota community of the three groups. **(E)** Phylogenetic tree and heatmap of abundance distribution between the groups. The left figure shows the evolutionary tree, in which the branches of different colors represent different gates, each branch at the end represents an operational taxonomic unit (OTU), and the end is annotated with the genus classification to which the corresponding OTU belongs. In the absence of corresponding genus classification, it is expressed as unclassied_genus, the heatmap on the right is standardized after the abundance, and the larger the value, the higher the relative abundance. **(F)** Association analysis between intestinal microbiome. * means significant, *** means extremely significant.

There was a total of 23 major bacterial genus in the three groups. The dominant bacterial families in the three groups of feces were Firmicutes, Bacteroidetes, Actinobacteria, and Proteobacteria (Figure 5A). The abundance of Firmicutes in FMT mice was higher than that in Ctrl mice, while the abundance of Bacteroidetes was lower than that in Ctrl mice. The ratio of Firmicutes to Bacteroidetes (F/B) of Ctrl mice (F/B = 0.32) was significantly (*P* < 0.01) lower than that of MSt mice (F/B = 0.50) and DLYt mice (F/B = 0.61). The abundance of Verrucomicrobia in Ctrl mice (3.68%) was higher than that in FMT mice (<0.3%). The most abundant bacteria in the three groups belong to *S24_7* (>45.63%)*, Lachnospiraceae* (>14.72%), and *Bacteroidaceae* (>10.12%) (Figure 5A). For instance, the predominant bacterial family in the MSt mice were *S24_7* (50.39%), *Lachnospiraceae* (18.83%), *Porphyromonadaceae* (5.41%), and *Coriobacteriaceae* (5.22%), while those of DLYt mice were *Unspecified_Clostridiales* (8.59%), *Ruminococcaceae* (7.15%), and *Rikenellaceae* (2.10%). Control mice had 3 bacterial families with higher abundance than the other two groups (Figure 5B). They were *Campylobacteraceae* (4.64%), *Porphyromonadaceae* (4.29%), and *Enterobacteriaceae* (4.01%).

The microbial taxonomy was analyzed by linear discriminant analysis effect size (LEfSe), with a log-linear discriminant analysis score > 4.0 and the Kruskal–Wallis test and the Wilcoxon rank sum test (*P* < 0.05) and it was found that there were statistical differences between control mice and FMT mice (Figure 5C). A total of 16 genera were significantly different among the three groups. In MSt mice, 7 genera including *Clostridiales* (*P* < 0.05), *Lachnospiraceae* (*P* < 0.05), and *Blautia* (*P* < 0.05) were significantly higher than the other groups. Three genera were significantly enriched in DLYt mice, including *Coriobacteriales* (*P* < 0.05). Six genera were significantly enriched in Ctrl mice, including *Bacteroides* (*P* < 0.05), *Acidifaciens* (*P* < 0.05), and *Bacidifaciens* (*P* < 0.05) (Figure 5D). Phylogenetic trees showed that the specific bacteria in MSt mice belong to the Bacteroides phylum, while which DLYt mice belong to Fusobacteria phylum and Ctrl mice had more microbiome belonging to Proteobacteria than those in FMT mice (Figure 5E). Most Firmicutes were negatively related to Bacteroides (Supplementary Figure 3). The majority of bacterial functions were mostly related to the cellular processes and environmental information processing of the host (Figure 5F). It showed that after the mice were subjected to cold stress, the function of the microbiota is mainly based on the processing of environmental information.

### Fecal Microbiota Transplantation Alters Mouse Short-Chain Fatty Acids Under Cold Exposure

**Figure 6:**
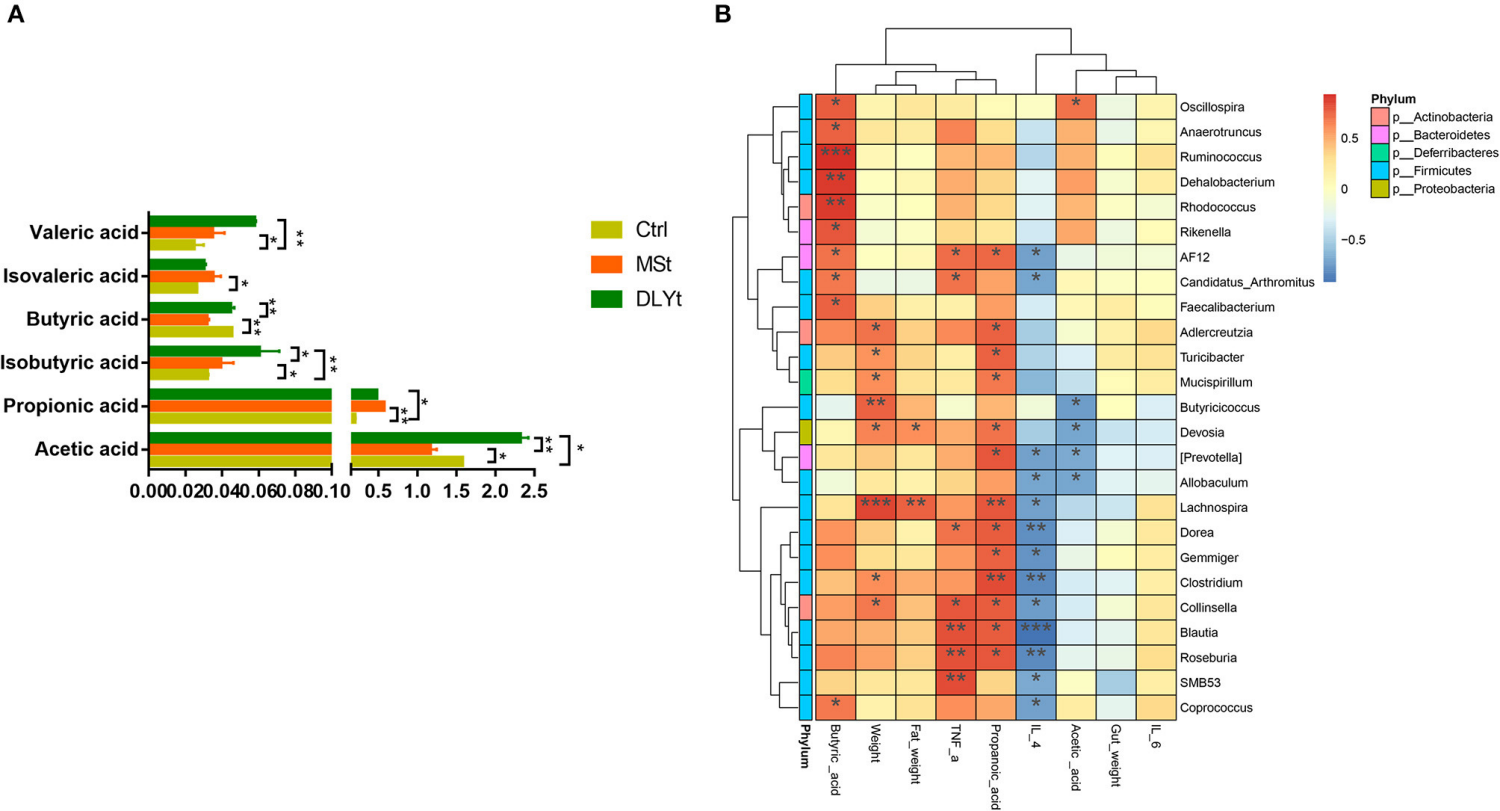
Effects of FMT on the concentrations of short-chain fatty acids during cold exposure. **(A)** Concentrations of acetic, propionic, isobutyric, butyric, valeric, and isovaleric acids of ileum (*n* = 7/group) (*P < 0.05, **P < 0.01, and ***P < 0.001, Duncan's multiple range test). **(B)** Heatmap of intestinal microbiota and relative functions index in the three groups. Heatmap displays Spearman's rank correlations between the relative abundance of bacterial genus and functional indicators concentrations across the fecal microbiota in the three groups. Only correlations with corrected values of *P* < 0.05 are illustrated. Blue edges represent negative correlation and pink edges represent positive correlation. The depth of color is proportional to the correlation coefficient (Monte Carlo rank test).

The DLYt mice had higher acetic acid, propionic acid, butyric acid, and valeric acid concentration than control mice during cold exposure, but decreased isobutyric acid (Figure 6A). The valeric acid content of Ctrl mice was significantly (*P* < 0.05) lower than that of MSt mice. The isovaleric acid content of MSt mice was significantly (*P* < 0.05) higher than that of Ctrl mice and the butyric acid content was significantly (*P* < 0.05) lower than that of Ctrl mice and DLYt mice. The content of isobutyric acid in DLYt mice was significantly higher than that in MSt mice, extremely significantly (*P* < 0.01) higher than that in Ctrl mice, and the content of isobutyric acid in MSt mice was significantly (*P* < 0.05) lower than that in DLYt mice. The content of propionic acid in MSt mice was significantly (*P* < 0.05) higher than that in Ctrl mice. The acetic acid content of MSt mice was significantly (*P* < 0.05) lower than that of Ctrl mice and DLYt mice. Interestingly, the contents of the *Blautia, Roseburia*, and SMB53 were positively associated with serum levels of TNF-α. We found that the content of propionic acid and butyric acid in rectal contents significantly changed and the abundances of *Clostridium* and *Lachnospira* showed significant correlations with changes in short-chain fatty acids. Furthermore, bacteria from the actinobacteria family were found to be positively correlated with butyric acid (Figure 6B).

## Discussion

Intestinal microbiota transfer has been shown to be more efficient, if the recipient's microbiota exhibits lower richness (37). But, residual antibiotics might persist in the mice and have deleterious consequences on the implantation of inoculum's bacteria. We, thus, subjected antibiotic-treated mice to bowel cleansing with PEG before. Previous study shows that microbiota engraftment performed better in younger mice (38). So, we chose young mice of 6 weeks. Germ-free (GF) rodents are the most commonly used receptor model because their gut lacks endogenous microorganisms and considering the lack of niche competition, they theoretically constitute a blank of experimental body (18). Microbiota transplantation can change the physiological functions of the host (39). The rectal temperature demonstrated that MSt mice were partly resistant to cold stress as shown and pig microbiota alone is sufficient to transfer part of this phenotype (Figures 1A,B). To reproduce niche competition similar to the GF model, most studies have exhausted recipient's initial gut microbiome with broad-spectrum antibiotics (10).

Pig adipose cannot produce *UCP1*, but in this study, fecal transplantation can increase the gene expression of the *UCP*1 gene in mice, indicating that fecal transplantation can still promote adipose browning in mice, but the specific mechanism still needs to be studied. Existing study has shown that *UCP*1 is essential for the protection of physical health under cold stress conditions (40). Acute cold exposure may enhance the induction of the expression of the canonical marker *PGC*-1α (41). MSt mice have high expression levels of UCP1 and PGC-1α. This implies increased adipose tissue browning in MSt mice (Figure 1C). FMT mice showed better thermogenic capacity. The adipose content of MSt mice is higher than that of DLYt mice and the high adipose content can improve the body's ability to keep warm, indicating that MSt mice have stronger fat storage ability. Leukocytes are an important component of the blood. High leukocyte counts may indicate that the immune system is working hard to destroy the infection, which is a normal immune response (42). Ctrl mice had more leukocytes than FMT mice, demonstrated that Ctrl mice enhanced the body's resistance to stress and immunity (Supplementary Figure 2). After the transplantation of fecal bacteria, a series of phenotypic characteristics and intestinal morphology of the mice have changed.

In our models, the number of goblet cells in MSt mice was significantly higher than that in Ctrl mice (Figure 2E). The ratio of villus height to recess depth can reflect the degree of renewal and metabolism of small intestinal epithelial cells (43). The increase of goblet cells can stabilize the intestinal barrier (44). Mice alleviate the effects of cold by increasing the turnover rate of small intestinal epithelial cells (45). The number of goblet cells in FMT mice is more than that in Ctrl mice, which is beneficial to increase the thickness of intestinal mucus in FMT mice, maintain intestinal barrier function, and reduce the infestation of pathogenic microbiome, while MSt mice have higher goblet cells, which prove that MSt mice had better gut health (Figures 2A–D).

Inflammatory cytokine results indicated stronger anti-inflammatory function in FMT mice. Interleukin-4 (IL-4) is the main cytokine involved in reducing the integrity of the epithelial barrier mediated by activated T helper 2 (Th2) cells (histamine and helper cells) (46). IL-4 is the main driver of mucosal barrier dysfunction, thereby overcoming the antagonism of TNF-α (47). The levels of IL-4 and TNF-α in MSt mice were reduced (Figure 3A). Cytokines, as signaling molecules between cells, play an important role in regulating inflammation, immune response, and tissue repair and their relative mRNA expression and serum content can reflect the health of the body (48–50). ZO-1, involved in maintaining and regulating epithelial barrier function, is often used as an indicator to observe the tight junction barrier function and permeability function of intestinal epithelial cells (51). Interestingly, reconstructing the intestinal microbiome from mice can effectively produce *IL*-15, *IL*-1, and *ZO*-1, indicating that inflammatory factors in FMT mice under cold stress play an important role under cold exposure and the intestinal barrier function of FMT mice is stronger and the change was more pronounced in MSt mice (Figures 3B,C). However, the expression of inflammatory factors in different intestines is different, which may be caused by functional differences in different intestines.

The diversity of gut microbiota in FMT mice was higher than that in Ctrl mice, demonstrating that fecal microbiota transplantation significantly changed the gut microbial structure of mice (Figures 4A,B). The fecal microbiota transplantation led to dramatic changes of the microbiota composition, increasing the ratio of Firmicutes to Bacteroidetes (Figure 5A). The relative abundances of Firmicutes and Bacteroidetes were relatively greater in the MSt mice. *Clostridiaceae* is a highly diverse family, encompassing genera that are important in nutrient digestibility (52). In this study, *Clostridiaceae* were enriched in MSt mice, suggesting that MSt mice have higher nutrient digestibility. Members of *Lachnospiraceae* are among the main producers of short-chain fatty acids and different taxa of *Lachnospiraceae* are also associated with different features. Their impact on the host physiology is often inconsistent across different studies (53). Coriobacteriales can be metabolized into volatile fatty acids, which have a strong correlation with energy metabolism (54). Different types of *Coriobacteriales* were enriched in DLYt mice, which played an important role for mice under cold conditions. Enrichment of *Bacteroidetes* is considered a marker of gut health. *Bacteroidetes*, as members of polysaccharide-degrading consortia, contribute to the release of energy from dietary fiber and starch, they are likely to be a major source of propionate, and they also have some activities that may help to suppress inflammation (55, 56). Our results show that *Bacteroides* play an important role in maintaining mouse health in Ctrl mice during cold exposure. The results of the microbiome abundance of FMT mice proved that the microbiome maintains intestinal balance and reshapes the intestinal environment (Figures 5B–F).

Short-chain fatty acids, especially butyrate, provide nutrients to the body, exert anti-inflammatory effects, and maintain intestinal homeostasis (57). Our results suggest that MSt mice have better anti-inflammatory ability (Figure 6A). In the liver, propionate inhibits *de-novo* lipogenesis and cholesterol production caused by acetic acid, making an increase in the ratio of propionic acid to acetic acid, an important determinant of the increase in lipid storage (58). Acetic acid can provide energy for the host (59) and the butyric acid and acetic acid contents of MSt mice were significantly lower than those of Ctrl mice and DLYt mice. The content of *Clostridium* and *Lachnospira* in MSt mice was related to the higher content of propionic acid. The propionic acid content of MSt mice was significantly higher than that of Ctrl mice and DLYt mice, proving that MSt mice may be have a stronger ability of *de-novo* lipogenesis and cholesterol production (Figures 6A,B). Studies are also needed to identify additional factors that influence the bacterial community, characterize other non-bacterial fractions of the pig microbiota, including fungi and viruses, and determine the relationship between cold exposure and microbiota composition.

## Conclusion

This study showed that fecal microbiota transplantation responds to cold-exposed mice and fecal microbiota transplantation effect of Mashen pigs is greater than that of DLY pigs. MSt mice gained more fat weight and reduced intestinal area during cold exposure. FMT alters gut morphology in cold-exposed environments of mice. The results of 16S rRNA-seq showed that FMT shaped the intestinal microbiota of mice by promoting the phylum Firmicutes in the feces and MSt mice changed the content of short-chain fatty acids, especially propionic acid, by increasing *Clostridium*. In summary, FMT not only changed the expression of inflammatory factors, but also changed the microbial composition and function and the effect was more obvious in MSt. These findings provide a reference for understanding the cold tolerance of different breeds of pigs. Future study may determine that certain bacterial species and metabolites were responsible for the mechanism of resistance to cold exposure conferred by FMT.

## Data Availability Statement

The datasets presented in this study can be found in online repositories. The names of the repository/repositories and accession number(s) can be found in the article/Supplementary Material.

## Ethics Statement

The animal study was reviewed and approved by the Code of Ethics of the World Medical Association (http://ec.europe.eu/environment/lab_animals/legislation_en.htm). Written informed consent was obtained from the owners for the participation of their animals in this study.

## Author Contributions

TL completed experiment, analyzed the data, and drafted the manuscript under the supervision of YY and XG. YG and CC carried out the bioinformatics analyses under the supervision of GC and BL. CL and PG conceived the study. All the authors read and approved the final version of the manuscript.

## Funding

This study was funded by the key research projects in the National Natural Science Foundation of China (Grant Number 31872336), the Program for Sanjin Scholar (Grant Numbers 2016, 2017), and the Basic Research Project of Shanxi Province (201901D211376 and 201901D211369).

## Conflict of Interest

The authors declare that the research was conducted in the absence of any commercial or financial relationships that could be construed as a potential conflict of interest.

## Publisher's Note

All claims expressed in this article are solely those of the authors and do not necessarily represent those of their affiliated organizations, or those of the publisher, the editors and the reviewers. Any product that may be evaluated in this article, or claim that may be made by its manufacturer, is not guaranteed or endorsed by the publisher.
