# Supplementary material for: Effect of Different Pig Fecal Microbiota Transplantation on Mice Intestinal Function and Microbiota Changes During Cold Exposure
Source: Front Vet Sci. 2022 Apr 13;9:805815. doi: 10.3389/fvets.2022.805815 (PMC9044030; doi:10.3389/fvets.2022.805815)
Supplement: Supplementary file 1 [file Data_Sheet_1.pdf]

Table S1 Quantitative primer sequence

	F	R
UCP1	CCGAAACTGTACAGCGGTCT	CCGAGAGAGGCAGGTGTTTC
Cpt1b	AATAAACCCCAGGATGGCGG	GGAGACGGACACAGATAGCC
Pgl-1α	GTGAAGAGATCAAGCCGGTCA	GTGTCCTGTCTCCGTGTAGC
IL-15	CAGAGGCCAACTGGATAGATG	ACTGTCAGTGTATAAAGTGGTGTCAAT
Zo-1	CGACAGTAGAACACGCTAT	AGAGGAAGGTATCAGAGGAG
IL-1	CATTCTGTCTCGAGCCCACC	GCTGGAAGTCTCTTGCGGAG
IL-10	ACTGCTAACCGACTCCTT	TCCACTGCCTTGCTCTTA

Table S2 Hematological values of mice during cold exposure

	Ctrl	MSt	DLYt
Leukocyte(10^9/L)	5.57±0.294a	4.017±0.941b	3.84±0.472b
Lymphocyte ratio (%)	71.3667±8.259	70.35±5.59	63.25±8.52
Intermediate cell ratio (%)	5.85±1.421	5.55±0.709	4.83±0.907
Granulocyte ratio (%)	21.78±7.199	24.65±4.528	31.3±9.981
Total number of red blood cells(10^12/L)	6.3467±0.726	6.825±0.254	6.955±0.526
Hemoglobin(g/L)	129±19.959	141.166±5.528	140.66±11.254
Hematocrit (fL)	34.933±4.115	37.66±1.779	38.26±2.941
Total number of platelets(10^9/L)	543.6667±5.65	548.833±4.418	538±7.249
Mean platelet volume (fL)	7.05±0.378	7.5333±0.307	7.7±0.876

Data are presented as means with their standard errors of the mean; n = 7 per group. Comparisons were conducted using one-way ANOVA test.

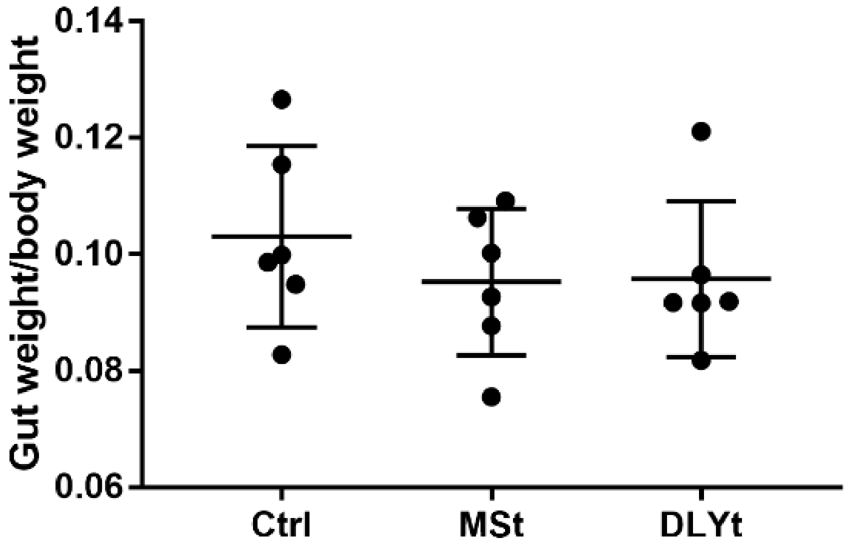

Figure S1 Gut weight/ body weight rate (n=7/group) in FMT and Ctrl mice during cold exposure.

Table S3 Hematological values of mice during cold exposure

	villus length（μm）		recesses depth（μm）		V/C	
	jejunum	ileum	jejunum	ileum	jejunum	ileum
Ctrl	411.38±13.11a	184.24±18.83a	103.13±12.10b	76.846±10.23	4.26±0.65a	2.23±0.34
MSt	232.19±16.92b	145.53±27.07b	124.74±16.92a	74.988±11.46	2.53±0.67b	1.98±0.65
DLYt	298.88±20.78b	156.67±12.06b	104.54±6.84b	79.205±9.82	2.90±0.34b	2.02±0.27

Data are presented as means with their standard errors of the mean; n = 7 per group.

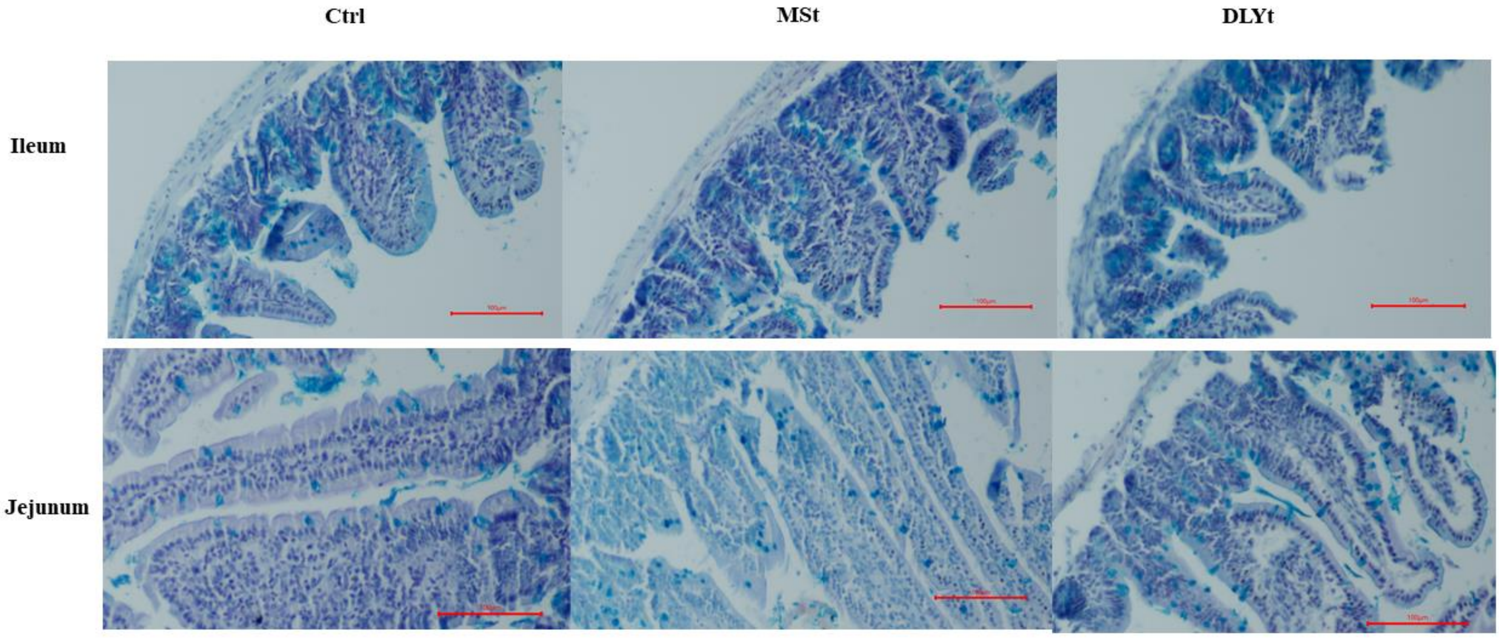

Figure S2 The ileum intestinal tissue AB_PAS stained sections of Control group , MSt group and DLYt group (n=7/group). The jejunum intestinal tissue AB_PAS stained sections of control group, MSt groupand DLYt group (n=7/group).

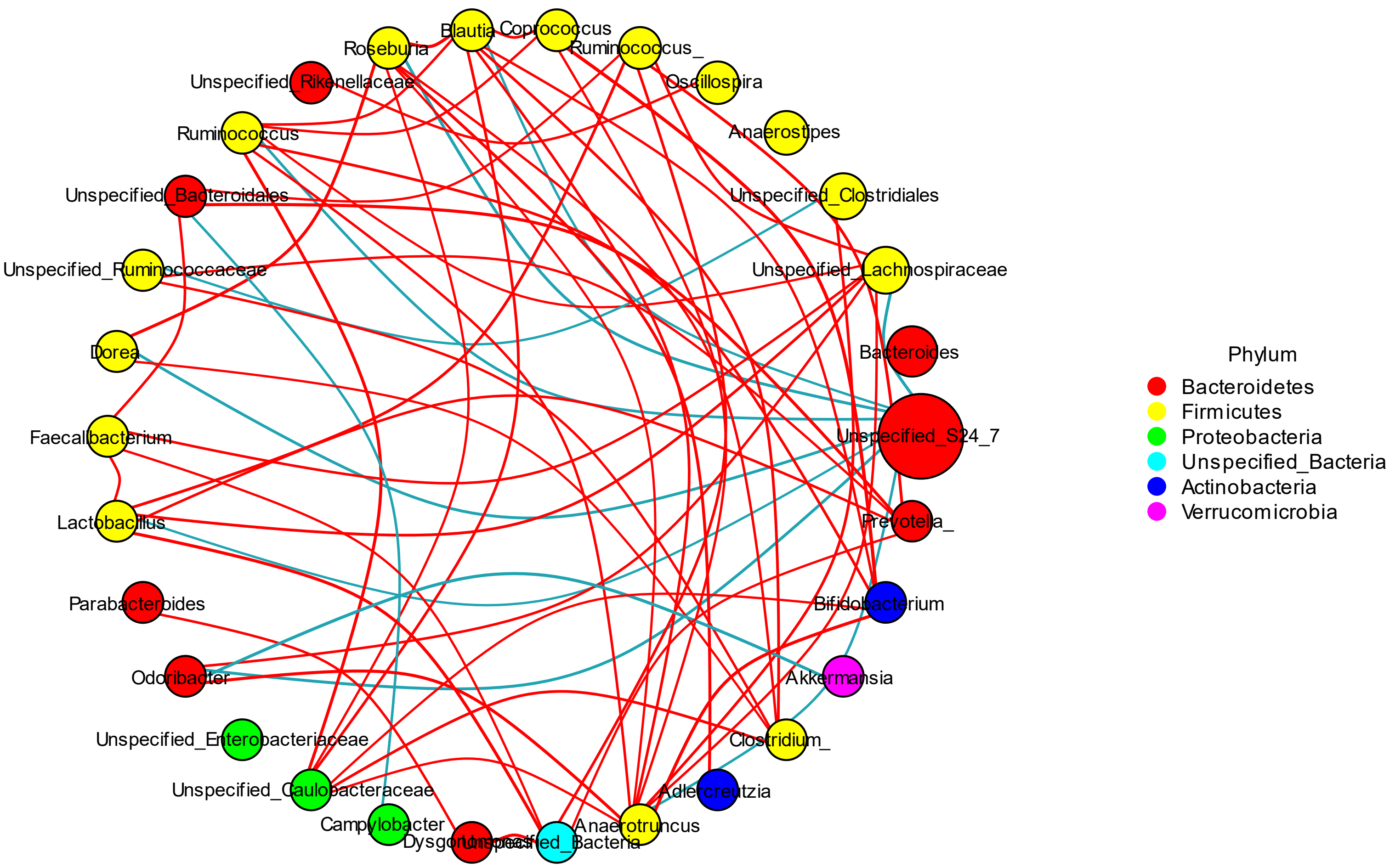

Figure S3 Genus classification level species interaction network diagram.

Comparison of the interaction network diagrams of the enriched species in the sample group. The circle represents a species, the size represents its relative abundance, the different colors represent different species phyla classifications, and the lines between the circles represent the two species. The correlation is significant ($P < 0.05$), the line color red represents positive correlation, blue represents negative correlation, the thicker the line, the greater the absolute value of the correlation coefficient.
